# Self-Assembled Oxide Nanoneedle-Induced Strain for Tunable Excitonic Emission in Monolayer MoS_2_

**DOI:** 10.1021/acsomega.6c04805

**Published:** 2026-10-02

**Authors:** Rashed H. Lone, Shreya Gaonkar, Deepak Sharma, Shruti Nirantar, E. S. Kannan

**Affiliations:** † School of Engineering, STEM College, 5376 RMIT University, Melbourne, VIC 3001, Australia; ‡ Department of Physics, BITS-Pilani K. K. Birla Goa Campus, Zuarinagar, Goa 403726, India; § Department of Electrical and Electronic Engineering, BITS-Pilani K. K. Birla Goa Campus, Zuarinagar, Goa 403726, India

## Abstract

Strain engineering has emerged as a powerful approach for tailoring the optical response of two-dimensional semiconductors. In this work, we show that monolayer MoS_2_ exhibits tunable excitonic emission, when nanoneedle-like oxide protrusions form across the monolayer surface during a photo-oxidation-assisted exfoliation process. These protrusions locally deform the lattice, generating spatially varying strain fields within the MoS_2_ layer. Photoluminescence and Raman measurements reveal a clear correlation between strained regions, exciton peak shifts, and enhanced emission, with the photoluminescence intensity increasing by approximately 3-fold, accompanied by a red shift of the in-plane (E_2g_^1^) vibrational mode, indicative of strain. Furthermore, selected monolayers and unexfoliated regions exhibit additional emergent near-infrared photoluminescence features absent in pristine MoS_2_. Unlike conventional strain-engineering approaches requiring external mechanical deformation, specialized substrate prepatterning, or complex heterostructures, this simple one-step process generates intrinsic nanoneedle-induced strain, providing an effective route for tailoring excitonic recombination and extending the optical response of monolayer TMDCs.

## Graphical abstract

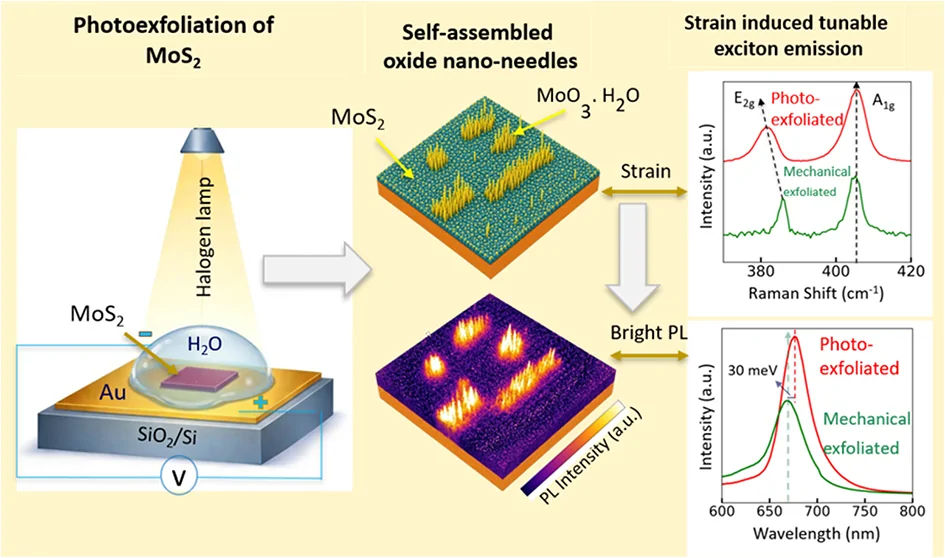

## Introduction

Monolayer (ML) transition metal dichalcogenides (TMDCs), such as MoS_2_, have emerged as prototypical two-dimensional (2D) semiconductors owing to their unique electronic, optical, and mechanical properties. Their direct bandgap, strong light–matter interactions, and large surface-to-volume ratio have enabled applications in electronic and optoelectronic devices, nanoelectromechanical systems (NEMS), and chemical and biological sensors.^1–4^ In addition, ML MoS_2_ exhibits pronounced excitonic effects arising from reduced dielectric screening, strong Coulomb interactions, and quantum confinement.^5–7^ In these atomically thin systems, tightly bound excitons dominate the optical response and underpin a broad range of photonic and optoelectronic applications, including ultrathin light emitters, photodetectors, modulators, and exciton-based information technologies.^8–10^ However, achieving robust and efficient excitonic photoluminescence (PL) in ML MoS_2_ remains challenging, as exciton recombination is highly sensitive to surface defects, charge disorder, and interactions with the local chemical and dielectric environment.^11–13^

Considerable effort has been devoted to enhancing excitonic emission in ML MoS_2_ and related TMDCs.^14–17^ Substrate engineering and dielectric screening have been employed to modify interfacial charge transfer and exciton recombination pathways. Choice of substrates and optimized dielectric environments can suppress nonradiative recombination and enable stronger exciton-dominated emission with improved PL quantum yield.^18–20^ Electrostatic gating and charge transfer doping provide control over the balance between neutral and charged excitons, enabling tunability of the optical response.^21,22^ In addition, chemical treatments and defect passivation strategies have been explored to improve emission intensity and spatial uniformity by reducing nonradiative recombination centers.^17,23^ Despite these advances, strategies for enhancing excitonic emission in pristine ML MoS_2_ through controlled surface modification remain relatively limited. While defect engineering and chemical functionalization have demonstrated substantial PL enhancement, the resulting optical response can depend sensitively on the local defect density, chemical environment, and treatment conditions, making spatially uniform and reproducible emission challenging in some approaches. Furthermore, to access longer-wavelength optical responses that are attractive for infrared photonic communication, sensing, and energy-efficient light-emitting technologies, often involve complex heterostructures, where interlayer coupling, moiré potentials, or type-II band alignment give rise to new excitonic states.^12,24–26^ Such strategies rely on precise stacking or interlayer interactions and do not originate from a single isolated ML.

Among the various approaches explored, strain engineering has emerged as a particularly powerful method for tailoring the optical properties of 2D TMDCs. Mechanical deformation of atomically thin layers can significantly modify electronic band structures, exciton binding energies, and recombination dynamics, enabling controlled tuning of excitonic emission energies and intensities.^27,28^ In ML MoS_2_, tensile strain is known to reduce the bandgap and induce red shifts in excitonic transitions, providing a route to engineer the spectral position of emission and potentially access near-infrared optical responses. However, most existing strain engineering strategies rely on externally applied mechanical deformation, patterned substrates, or complex device architectures, which can limit scalability and reproducibility.^27–29^ Developing simple and intrinsically generated strain fields within ML materials therefore remains an important challenge.

Here, we demonstrate that a photo-oxidation-assisted exfoliation process yields ML MoS_2_ decorated with nanoneedle-like oxide protrusions distributed across the ML surface. These nanoscale structures generate localized tensile strain in the underlying lattice, leading to spectral tuning and strong enhancement of excitonic PL. PL and Raman measurements reveal a clear correlation between nanoneedle protrusions and enhanced emission, accompanied by systematic shifts in exciton peaks and vibrational signatures consistent with strain in the ML. Power-dependent Raman measurements further reveal the dynamic and surface-confined nature of these nanostructures. In addition, selected MLs and unexfoliated regions exhibit emergent near-infrared PL features not observed in pristine MoS_2_, indicating the formation of additional recombination pathways associated with the nanoneedle-induced strain. These findings demonstrate that nanoneedle-induced strain provides an effective route for engineering excitonic emission and enabling near-infrared optical responses in MoS_2_, eliminating the need for external deformation, specialized substrate prepatterning, or complex heterostructures, and highlighting the potential of controlled surface morphology for next-generation 2D optoelectronic devices.

## Experimental Section

Bulk MoS_2_ crystals (HQ Graphene) were mechanically exfoliated (ME) using Nitto tape to obtain thin flakes with typical thicknesses of ∼50–100 nm. The exfoliated flakes were first transferred onto a poly­(dimethylsiloxane) (PDMS) stamp and subsequently aligned on the target substrate using a transfer stage equipped with an optical microscope before being brought into contact with a precleaned SiO_2_/Si substrate (300 nm SiO_2_) coated with a 100 nm Au layer deposited by electron-beam evaporation (Figure 1). Gentle pressure was applied to promote adhesion of the flakes to the Au surface, after which the PDMS stamp was slowly peeled away, leaving the MoS_2_ flakes on the substrate for subsequent characterization and measurements. The Au layer served as a conductive back electrode during the thinning process. ML MoS_2_ was obtained via a bias-assisted photoexfoliation (PE) process. Deionized water was drop-cast onto the flake region, followed by uniform illumination using a broadband halogen light source (455–660 nm) while a +3 V bias was applied to the Au substrate for approximately 3 min.

**Figure 1:**
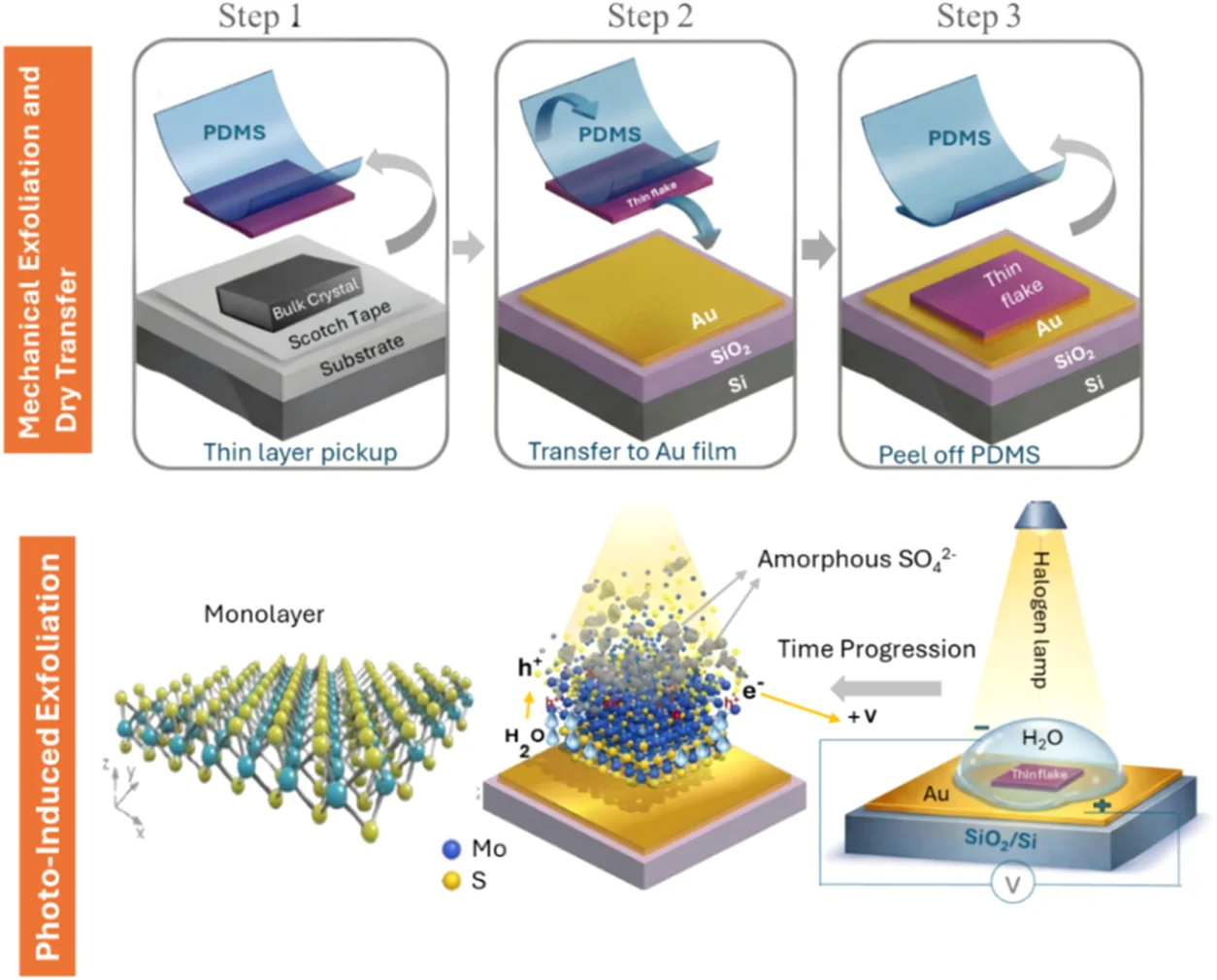
Schematic of the exfoliation and transfer workflow. Mechanical exfoliation of bulk crystals using a PDMS stamp produces thin flakes (step 1), which are subsequently dry-transferred onto target substrates (steps 2 and 3). The flakes are then subjected to PE under a 3 V bias applied via the conductive substrate for 3 min under broad-spectrum illumination (455–660 nm). Simultaneously, deionized water is precisely drop-cast onto the flake-covered region, facilitating the formation of MLs.

Following the thinning process, samples were thoroughly rinsed with deionized water and dried under a nitrogen flow. Surface morphology and thickness were examined using atomic force microscopy (Bruker Dimension Icon AFM) using a Scan Asyst-air tip operated in tapping mode to confirm ML thickness and to identify oxide-related surface features. Raman scattering and PL measurements were performed using a Horiba LabRAM HR Evolution spectrometer with a 532 nm excitation laser focused through a 100× objective (NA = 0.9), resulting in a spot size of approximately 0.7–1 μm. All measurements were conducted at room temperature under low laser power (∼2.5 mW) to minimize local heating effects, unless otherwise specified. Spectra were acquired with identical acquisition parameters (integration time, accumulations, and laser power) for all samples. Measurement locations were selected based on optical contrast and PL mapping, with optically lighter regions used to confirm monolayer thickness and optically darker regions exhibiting enhanced PL probed to investigate strain-related effects associated with the nanoneedle-like protrusions. For direct intensity comparison between ME and PE monolayers, raw (unnormalized) spectra are presented. Both ME and PE samples were measured on identical Au/SiO_2_/Si substrates, ensuring that any surface-enhanced Raman scattering (SERS) effects from the Au layer influence both data sets similarly.^30,31^ Peak positions were determined by identifying the maximum intensity point. Power-dependent PL and Raman measurements were conducted to probe excitation-induced changes in exciton recombination pathways and to examine laser-induced modifications of surface morphology, respectively. The oxidation states of molybdenum before and after exfoliation process were analyzed using X-ray photoelectron spectroscopy (Kratos AXIS Supra XPS spectrometer with a 1486.7 eV (Al Kα) X-ray source) under ambient conditions.

## Results and Discussion

In PE process under the illumination, photoexcited carriers are generated in MoS_2_, and the applied bias promotes charge separation across the MoS_2_–Au interface. Electrons are efficiently extracted into the Au substrate, leaving holes localized at defect sites (edges, vacancies, and steps), where they promote oxidation of the upper layers of MoS_2_ through the chemical reaction shown in eq 1 ^32^

1 $${MoS}_{2}+{11H}_{2}O+{18h}^{+}\to {MoO}_{3}+{2SO}_{4}^{2-}+{22H}^{+}$$

The oxidation proceeds in a spatially heterogeneous and self-limiting manner. The oxidized MoO_3_ species is weakly bound and partially soluble in water, undergoing dissolution as described in eq 2

2 $${MoO}_{3}+H_{2}O⇌{MoO}_{4}^{2-}+{2H}^{+}$$

This repeated oxidation–dissolution cycle preferentially removes the upper layers of the multilayer flake, ultimately yielding ML MoS_2_ while preserving the underlying lattice. The self-limiting nature of the PE process is attributed to two factors.^33^ Upon reaching the monolayer limit, the large exciton binding energy of direct-bandgap MoS_2_ suppresses charge separation, thereby reducing the availability of free holes required for further photo-oxidation. Additionally, strong interfacial coupling between the monolayer and the underlying Au substrate stabilizes the lattice against further removal. Consequently, the exfoliation process terminates at the monolayer thickness while the overlying layers are selectively removed.

To understand the optical, vibrational, and structural evolution of MoS_2_ during the PE process, a comprehensive analysis confirming the successful conversion of bulk flakes into ML MoS_2_ is presented (Figure 2). Figure 2a and its insets show optical microscopy images of two MoS_2_ flakes before and after the exfoliation process, respectively. Prior to exfoliation (Figure 2a), the flake exhibits strong optical contrast with respect to the underlying gold film which is a characteristic of a thick, multilayer or bulk region. After exfoliation (insets), the same region displays a nonuniform, faint contrast consistent with ML MoS_2_, indicating substantial thickness reduction of approximately 90% of the total surface area without lateral fragmentation. The preservation of the lateral dimensions highlights the self-limiting nature of the thinning process and indicates minimal lateral etching or fragmentation of the flakes, distinguishing it from more aggressive chemical or plasma-based thinning approaches. Although preservation of flake morphology alone does not preclude the formation of local defects, the well-defined Raman and photoluminescence characteristics observed subsequently (Figure 2b,2c) suggest that the monolayers retain good optical quality after the thinning process. Figure 2b compares the Raman spectra of the flake before and after exfoliation, focusing on the characteristic in-plane E_2g_^1^ and out-of-plane A_1g_ phonon modes. In the exfoliated region, the frequency separation (19.13 cm^–1^) between these two modes is reduced compared to the bulk flake (25.15 cm^–1^), consistent with the thickness-dependent Raman evolution toward ML MoS_2_. This reduction in peak separation is a well-established fingerprint of the transition from multilayer to ML thickness, confirming that the exfoliation process effectively yields a single MoS_2_ layer.^34^ Figure 2c compares the PL spectra of the flake before and after exfoliation. The bulk MoS_2_ region exhibits weak PL almost invisible, due to its indirect bandgap nature, where nonradiative recombination pathways dominate. In stark contrast, strong PL emission was observed in ML MoS_2_, reflecting its transition to a direct bandgap nature and the increased radiative recombination efficiency of excitons. From these optical signatures, it is quite evident that PE process reliably produces high-quality MLs of MoS_2_.

**Figure 2:**
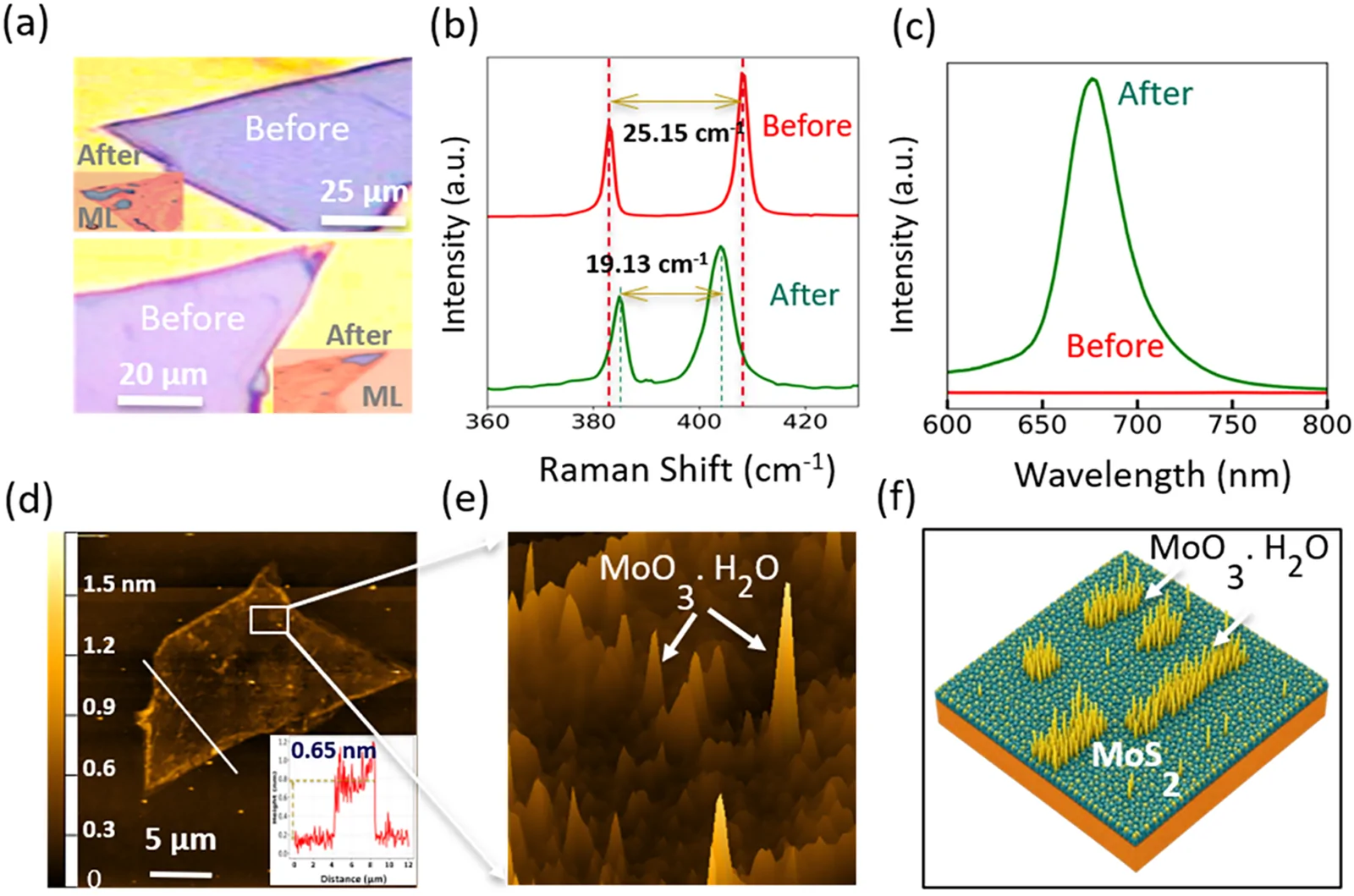
(a) Optical micrographs of two MoS_2_ flakes before and after (insets) the exfoliation process. (b) Raman spectra exhibiting reduced E_2g_^1^–A_1g_ mode separation after PE process, characteristic of ML MoS_2_. (c) PL spectra before and after PE demonstrating the indirect-to-direct bandgap transition from bulk to ML. (d) AFM topography image and corresponding height profile (inset) showing an average thickness of ∼0.65 nm, consistent with ML MoS_2_. (e) 3D magnified AFM image taken from the region highlighted (white box) in Figure 2d, highlighting the formation of nanoneedle-like oxide structures. (f) Schematic illustration of the ML, depicting the formation of localized oxide protrusions.

The thickness of the exfoliated region is directly quantified using AFM. Figure 2d and its inset present AFM topography across the exfoliated region and the corresponding height profile. The measured step height of approximately 0.65 nm is in excellent agreement with the reported thickness of ML MoS_2_, further validating the formation of a ML.^35^ AFM height profile reveals modest surface roughness attributed to thin, spatially inhomogeneous residual oxide species formed during exfoliation. Although this oxide-related roughness is weak across most of the flake surface and largely averaged out in the thickness profile, more pronounced morphological features are observed locally on the surface of ML. Figure 2e is the 3D magnified AFM image taken from the region highlighted in Figure 2d which highlights the structural manifestation of these pronounced morphological features as nanoneedle-like protrusions formed on the ML surface. Similar protrusions have previously been reported by Zhang et al.^36^ It is worth noting that a continuous boundary is observed at the edges of the ML (Figure 2d), which is most likely due to the accumulation of amorphous oxide species along with the formation of oxide protrusions during the PE process. AFM height profile (Figure 2d) indicate that the effective ML thickness is preserved despite the presence of these surface protrusions, confirming that the oxide species exist as localized nanostructured protrusions rather than forming a continuous overlayer. The schematic in Figure 2f illustrates the spatial distribution of these protrusions, depicting an exfoliated MoS_2_ ML decorated with nonuniform nanoneedle-like protrusions. These protrusions are formed as dissolved molybdate species locally recrystallize at reactive sites, such as intrinsic defects and flake edges, into stable hydrated molybdenum oxide nanostructures (eq 3), which appear as nanoneedle-like protrusions.

3 $${MoO}_{4}^{2-}+{2H}^{+}+H_{2}O\to {MoO}_{3}\cdot H_{2}O$$

Sulfur released during oxidation forms soluble SO_4_^2–^ ions that remain in solution and do not participate in nanocrystal formation. Overall, these results provide the structural and optical foundation for the subsequent analysis of exciton dynamics in PE MLs.

AFM topography was used to quantitatively characterize the nanoneedle structures formed on the PE MoS_2_. A representative 2D AFM image of Sample 1 and two line profiles (P1 and P2) are shown in Figure 3(a–c), with the height and lateral width of individual structures determined directly from the corresponding profiles. Measurements were performed on two regions of each of three independently analyzed samples. The resulting height and width distributions are summarized in Figure 3(d,e), with individual measurements (open circles) shown together with the mean (bars) ± SD (error bars). The nanoneedles exhibit characteristic heights of approximately 5–19 nm and lateral widths of 0.1–0.6 μm, with some sample-to-sample variation. Additional AFM images and corresponding line-profile measurements from Samples 2 and 3 are provided in the Supporting Information (Figure S1). The AFM topography was further analyzed using a height threshold of approximately 2–5 nm to distinguish the pronounced protrusions from the underlying MoS_2_ surface roughness. The nanoneedle coverage was calculated from the projected area of the thresholded regions according to, $C_{\mathrm{NN}}=\left(\frac{A_{\mathrm{NN}}}{A_{{\mathrm{MoS}}_{2}}}\right)\times 100\%$ , where *A*_NN_ is the projected area of the thresholded nanoneedle regions and *A*_MoS_2__ is the total analyzed MoS_2_ area. The resulting nanoneedle coverage was 27.23%, 31.78%, and 43.02% for Samples 1, 2, and 3, respectively. A representative height-thresholded AFM image for Sample 1 is provided in Figure S2 in the Supporting Information.

**Figure 3:**
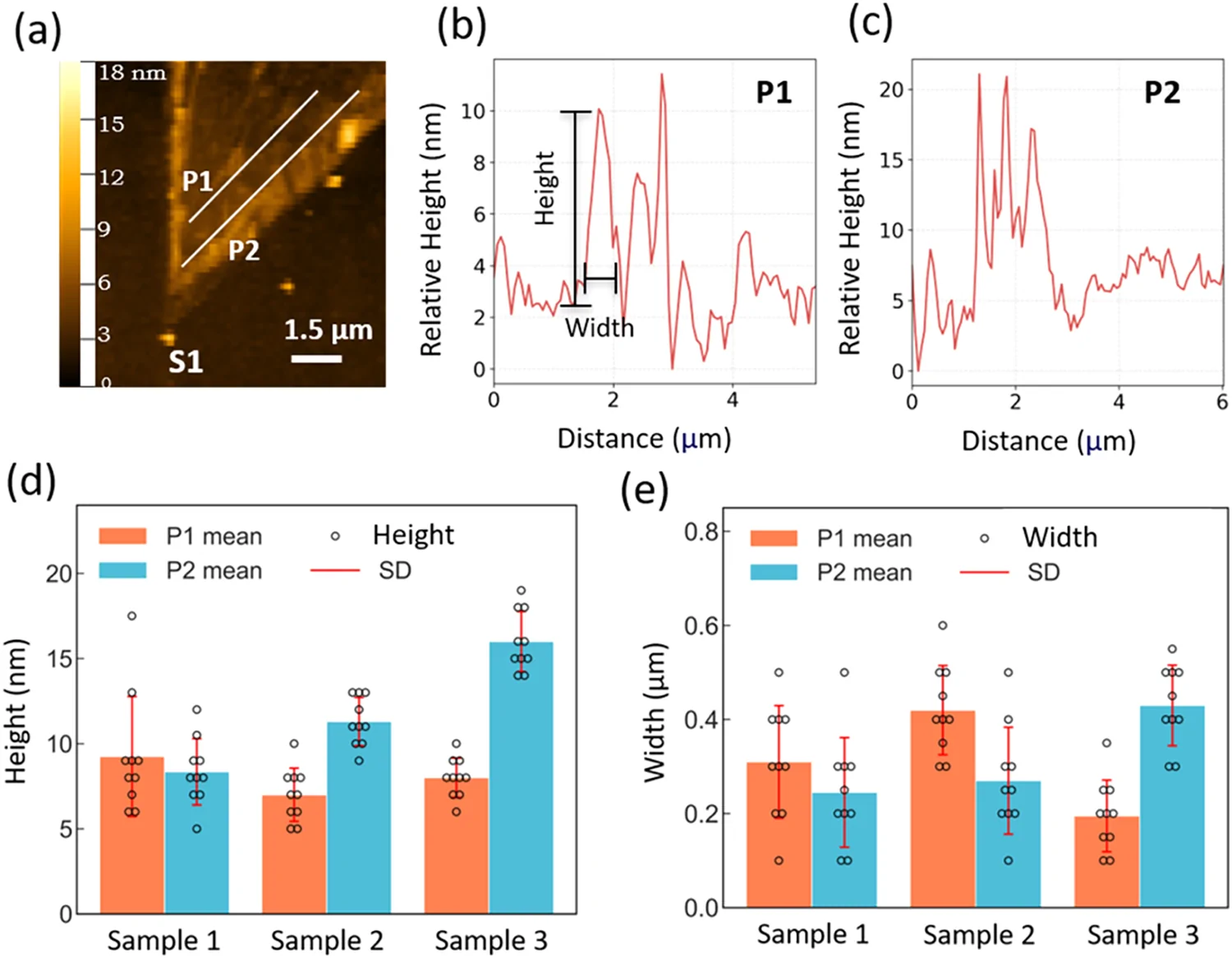
(a) AFM topographic image of Sample 1 showing the two selected line-profile regions, P1 and P2. (b, c) Corresponding height profiles along P1 and P2, respectively. The black markers in (b) illustrate the method used to determine the nanoneedle height and lateral width from the line profile. (d, e) Statistical analysis of the nanoneedle height and width, respectively, obtained from P1 and P2 measurements for Samples 1–3. Open circles represent individual measurements, bars represent the mean values, and error bars indicate the standard deviation (SD).

To understand the effect of PE on exciton dynamics, a comparative analysis of PL and Raman spectra of MLs obtained via direct ME and the PE process was carried out. Figure 4a presents the PL spectra of PE ML containing nanoneedles and ME ML on an Au-coated SiO_2_ substrate. The PE ML exhibits significantly stronger PL emission compared to the ME ML, along with a red shift in the PL peak position of approximately 30 meV relative to the ME ML. Spatially resolved PL mapping (Figure 4b) of the PE MoS_2_ ML reveals a pronounced enhancement in PL emission across the ML, except in the unexfoliated bulk region. In contrast, the PL map of the ME ML on Au (Figure 4c) shows comparatively lower PL emission intensity across the entire ML. To understand the physical mechanism behind the enhanced PL characteristics of PE MLs, PL emission of PE MLs is compared with that of ME MLs on Au and SiO_2_ substrates. A clear hierarchy in PL intensity is observed, with the PE ML exhibiting the strongest emission, followed by the ME ML on Au/SiO_2_ and then ME ML on SiO_2_. The enhanced emission from ME ML MoS_2_ on Au relative to SiO_2_ is accompanied by a ∼30 meV blue shift, which can be attributed to substrate-induced charge redistribution at the MoS_2_–Au interface.^20–22,37,38^ Differential work function induced charge transfer from MoS_2_ to Au depletes intrinsic n-type carriers, shifting the dominant excitonic recombination pathway from low-yield charged trions (A^–^) to highly radiative neutral excitons (A^0^), thus 30 meV blueshift. In contrast, SiO_2_ substrates typically host a high density of trapped charges and surface states that promote nonradiative recombination and exciton quenching, thereby limiting the PL intensity of ML MoS_2_.^39,40^ Although intimate contact with metallic substrates typically induces PL quenching through nonradiative energy transfer, ME monolayers can retain enhanced PL when nanoscale separation between the MoS_2_ and Au substrate is introduced by surface adsorbates and substrate roughness. Such interfacial nanoscale gaps of a few nanometres can reduce the short-range nonradiative energy transfer to the metal and thereby suppress short-range nonradiative PL quenching.

**Figure 4:**
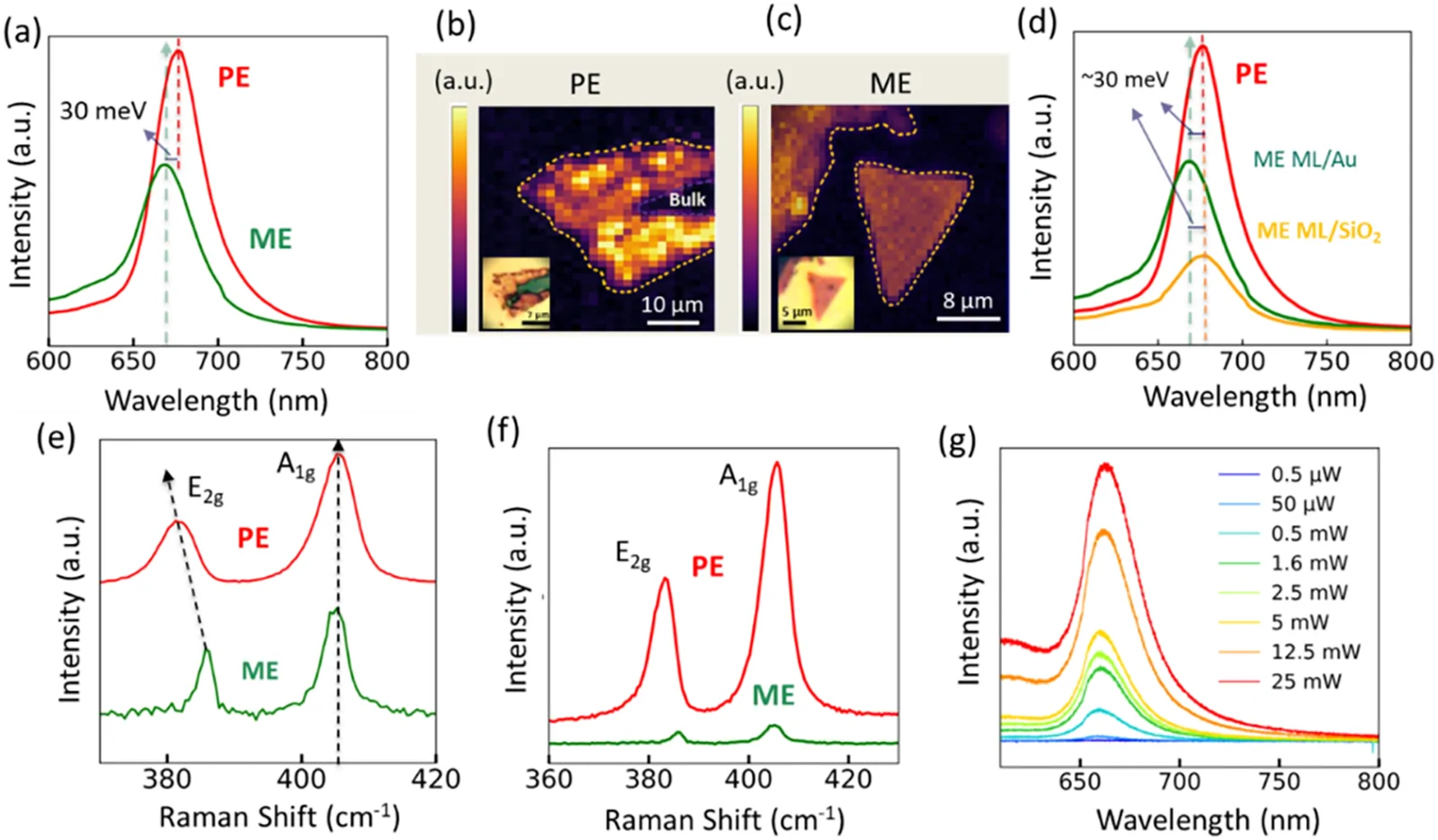
(a) Comparative PL spectra of PE (red) and ME (green) MoS_2_ MLs on an Au substrate. (b) PL intensity map of a PE MoS_2_ ML on Au, with the corresponding optical image (inset). (c) PL intensity map of an ME MoS_2_ ML on Au, with the corresponding optical image (inset). (d) Comparative PL spectra of PE and ME MoS_2_ MLs on Au and SiO_2_ substrates. (e) Normalized Raman spectra comparing peak positions of PE and ME MoS_2_ MLs. (f) Raman intensity comparison between PE and ME MoS_2_ MLs. (g) Power-dependent PL spectra of a PE MoS_2_ ML.

The pronounced PL enhancement, together with the red shift of the A^0^ peak in the PE ML, indicates the presence of an additional mechanism beyond substrate-induced effects. The observed optical characteristics suggest a combined effect of the conducting Au substrate, (via charge transfer) and hydrated molybdenum oxide nanostructures (MoO_3_·H_2_O) (via strain and charge transfer). These hydrated oxide protrusions on the ML induce tensile strain in the underlying lattice, which modifies the electronic band structure, effectively altering the bandgap, as evidenced by an ∼30 meV redshift in the PL peak. This strain may promote exciton localization and suppress diffusion to nonradiative recombination centers, while also enhancing exciton–phonon coupling that facilitates relaxation into radiative states, collectively increasing the probability of radiative recombination and resulting in enhanced PL intensity. Because the top-surface nanoneedles (MoO_3_·H_2_O) are directly coupled to the MoS_2_ surface, they act as electron acceptors, inducing complementary p-doping and modifying the relative populations of neutral (A^0^) and charged (A^–^) excitons, while the associated strain promotes exciton funneling. These effects can collectively enhance radiative exciton recombination. Thus, the observed PL enhancement is attributed to the combined effects of nanoneedle-induced tensile strain, (MoO_3_·H_2_O)–MoS_2_ interfacial charge transfer, and Au–MoS_2_ charge transfer, with a possible additional contribution from the modified local dielectric environment.

Previously, strain engineering has been widely utilized to modulate the electronic band structure of 2D TMDCs, enabling tunable bandgaps and modified carrier dynamics.^27–29,41–43^ This interpretation is supported by Raman spectroscopy (Figure 4e), where a systematic red shift of the in-plane E_2g_^1^ mode is observed in PE ML (containing nanoneedles) relative to ME ML, while the out-of-plane A_1g_ mode remains unchanged. Because the E_2g_^1^ vibration involves in-plane atomic motion, its softening indicates tensile lattice distortion induced by the oxide domains, whereas the stability of the A_1g_ mode suggests that vertical lattice symmetry remains largely preserved. It should be noted that the Raman spectra in Figure 2b and Figure 4e were acquired from different samples. The spectrum in Figure 2b corresponds to a nanoneedle-free ML region, whereas that in Figure 4e was obtained from a ML region containing the nanoneedle structures. Therefore, the PE monolayers presented in Figure 2a–c serve as a control sample for evaluating the effects associated with the oxide nanoneedle structures. In addition to strain, the oxide protrusions may contribute to secondary effects such as local dielectric modification, and passivation of sulfur vacancies, which may further suppress nonradiative recombination and enhance neutral exciton emission.^17,19,44–52^ Previous studies have reported that interactions between monolayer MoS_2_ and Au substrates can modify Raman and photoluminescence characteristics through charge transfer and dielectric screening effects.^53–55^ However, in the present work, both the PE and ME monolayers were investigated on the same Au substrate. Notably, neither the ME monolayer nor the optically lighter PE monolayer regions (without nanoneedles) exhibit a measurable red shift of the E_2g_^1^ mode. In contrast, the optically darker PE regions containing nanoneedle-like structures show both a red-shifted E_2g_^1^ mode and enhanced PL emission. This spatial correlation indicates that the observed spectral changes are predominantly associated with nanoneedle-induced local strain, and the charge redistribution (MoO_3_·H_2_O–MoS_2_) rather than substrate interactions alone. Additional insight is provided by the Raman intensity analysis shown in Figure 4f. The Raman intensities of both E_2g_^1^ and A_1g_ modes increase significantly in the PE ML compared to the ME ML. Strain-induced modulation of electron–phonon coupling can enhance the Raman scattering cross-section, leading to stronger vibrational responses.^41^ Importantly, the well-defined Raman peaks indicate that the oxide protrusions induce controlled lattice distortion while preserving the crystalline integrity of the underlying ML. Power-dependent PL measurements taken from PE ML (Figure 4g), shows a monotonic increase in emission intensity with increasing excitation power without observable saturation. The absence of significant quenching at higher excitation densities indicates that radiative excitonic recombination remains dominant and that nonradiative processes such as defect-assisted recombination or exciton–exciton annihilation do not significantly limit emission under the measured conditions.

Grain boundaries in MoS_2_ are known to introduce localized strain fields and defect states that can modify excitonic recombination, carrier transport, and optical emission.^56,57^ However, these effects are unlikely to contribute in the present work, as the starting material consists of single-crystalline MoS_2_ flakes and the exfoliation process involves thickness reduction rather than crystal growth. Therefore, the Raman and photoluminescence characteristics reported here are attributed to local lattice deformation induced by these oxide nanoneedles rather than grain-boundary effects. Together, these spatially resolved optical and vibrational measurements demonstrate that oxide nanoneedle-induced strain acts as a localized exciton-engineering mechanism in ML MoS_2_. The strong correlation between oxide protrusions, exciton peak shifts, E_2g_^1^ phonon softening, and enhanced excitonic emission identifies tensile strain as the primary driver of exciton modulation. This behavior highlights the critical role of surface morphology in modulating the excitonic landscape of ML MoS_2_ and provides a simple route for enhancing radiative recombination without relying on complex heterostructures or external electrostatic gating.

In selected PE MLs and PE treated unexfoliated regions, several prominent low-energy PL peaks were observed as shown in Figure 5a. The spectra plotted in green correspond to PE MLs, while the spectra plotted in orange correspond to PE treated unexfoliated regions. In addition to the characteristic direct band gap emission at ∼1.85 eV, selected PE MLs exhibit an additional emission peak at ∼1.56 eV. PE-treated unexfoliated regions display multiple low-energy emission features, including peaks at approximately 1.75, 1.56, 1.43, and 1.29 eV, which are associated with regions retaining residual multilayer material and/or oxide structures. To evaluate the reproducibility of these observations, a total of 13 PE-treated flakes were investigated. Among these, eight fully exfoliated monolayer flakes consistently exhibited the characteristic direct-bandgap excitonic emission near ∼1.85 eV. However, the additional ∼1.56 eV emission was observed in only three of these monolayers, specifically in localized oxide nanoneedle-rich regions. In contrast, all five incompletely exfoliated flakes containing residual oxide structures exhibited additional emission features spanning ∼1.29–1.75 eV. These results demonstrate that the PE process reproducibly yields monolayer MoS_2_ with the characteristic direct-bandgap excitonic emission, whereas additional sub-bandgap emission features occur selectively in certain nanoneedle-rich regions and in incompletely exfoliated regions. The recurrent ∼1.56 eV emission in both fully and incompletely exfoliated regions indicates that it is not uniquely associated with either monolayer or residual multilayer material; its microscopic origin therefore remains unresolved from the present measurements, and no specific electronic transition or defect assignment is made. In contrast, the ∼1.29 eV feature, observed exclusively in incompletely exfoliated regions, is consistent with reported indirect-gap emission from multilayer MoS_2_ arising from thickness-dependent electronic structure and interlayer coupling. The weaker ∼1.43 and ∼1.75 eV features occur inconsistently across the examined regions. Although emission near ∼1.75 eV has been associated with defect-related excitonic states, including sulfur-vacancy-related states, the present measurements do not identify the responsible defect species and therefore do not support a specific assignment. The origin of the ∼1.43 eV feature likewise remains unresolved. Overall, the additional sub-bandgap emissions are not attributed to a single mechanism but are associated with the local structural state of the PE-treated material, with the ∼1.29 eV feature being consistent with residual multilayer MoS_2_, while the origins of the ∼1.43, ∼1.56, and ∼1.75 eV features require further investigation. Figure 5b compares the PL spectra of a ME ML MoS_2_ (red) with that of a PE ML MoS_2_ (green). In this comparison, the ME ML exhibits a distinct B exciton peak, whereas the B exciton feature is absent in the PE ML spectrum. This suppression reflects a modification of the local electronic environment, likely arising from effects such as strain, interfacial charge redistribution, sulfur vacancy passivation or altered dielectric screening. These effects may reduce the oscillator strength and population of the B exciton without affecting the dominant A exciton. These results underscore surface morphology as a highly effective and localized tuning parameter for excitonic engineering in 2D semiconductors, capable of simultaneously enhancing *A*-exciton emission while reconfiguring novel near-infrared excitonic transitions depending on the local structural and electrostatic environment.

**Figure 5:**
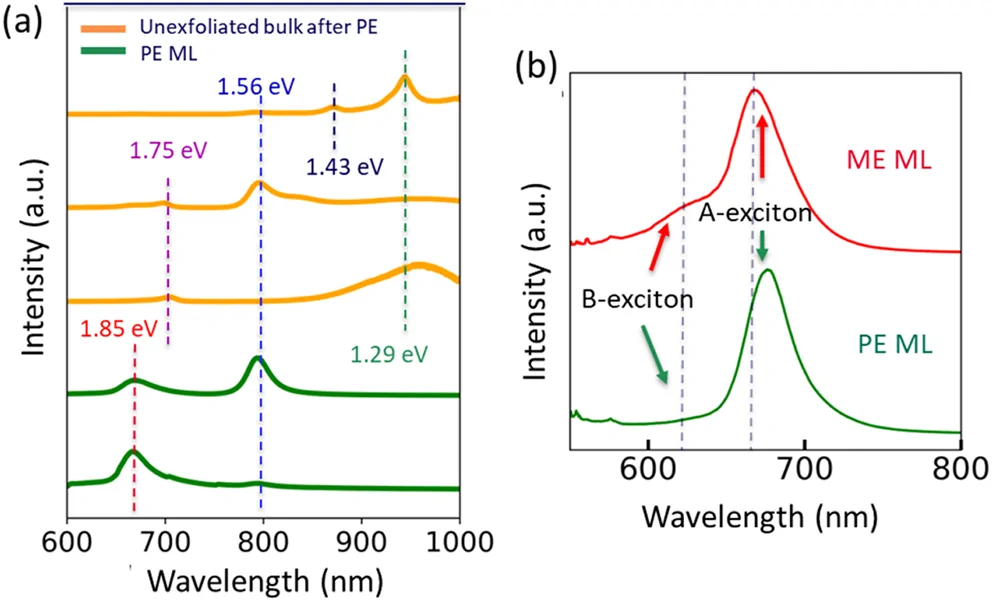
(a) PL spectra acquired from a few selected flakes, where the green curves represent PE MLs and the orange curves correspond to unexfoliated regions following PE. (b) Comparative PL spectra of a ME ML (red) and a PE ML (green), highlighting the presence of the B exciton in the ME sample and its suppression in the PE ML.

To understand the chemical changes induced by the PE process, XPS analysis of the MLs was performed before and after PE. Figure 6a,b present the Mo 3d core-level XPS spectra of MoS_2_ flakes acquired before and after PE, respectively. The Mo 3d XPS spectra reveal a clear evolution of the molybdenum oxidation state after exfoliation. Compared to the pristine flake, which exhibits only the characteristic Mo^4+^ doublet, the exfoliated ML shows additional higher–binding-energy components corresponding to oxidized molybdenum species (Mo^6+^).^52^ The Mo^4+^ contribution is represented by the spin–orbit split Mo 3d doublet with Mo 3d_5/2_ and Mo 3d_3/2_ peaks centered at ∼229.5 eV and ∼232.6 eV, respectively. The oxidized Mo^6+^ state is similarly fitted using a spin–orbit doublet with Mo 3d_5/2_ and Mo 3d_3/2_ components located at ∼232.8 eV and ∼235.9 eV. During peak fitting, the spin–orbit splitting for each doublet was constrained to ∼3.1 eV, with a fixed area ratio of 3:2 between the 3d_5/2_ and 3d_3/2_ components, consistent with the degeneracy of the Mo 3d states. Additionally, the full width at half-maximum (fwhm) was constrained to be identical for the two peaks within each doublet to ensure physically consistent line shapes. The coexistence of Mo^4+^ and Mo^6+^ indicates that ML MoS_2_ remains intact after PE process along with the presence of Mo-oxide species. Collectively, the XPS and AFM results demonstrate that PE couples layer thinning with controlled surface-confined formation of oxide nanostructures, producing optically modified MLs without compromising the underlying lattice integrity.

**Figure 6:**
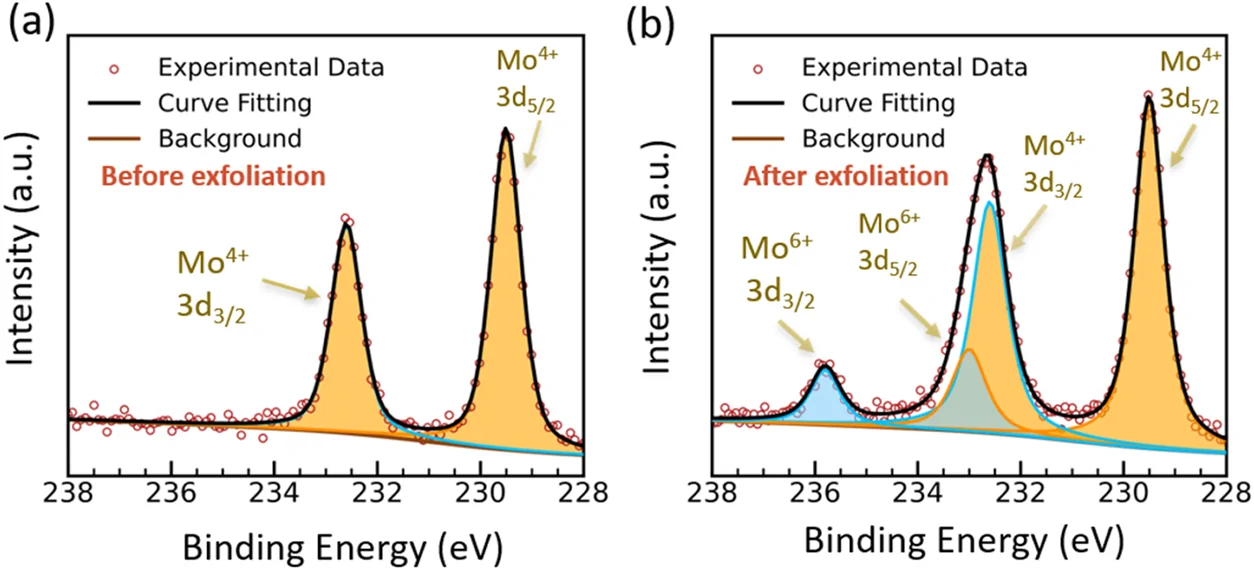
(a) Mo 3d XPS spectrum acquired prior to PE, showing the characteristic Mo^4+^ doublet of MoS_2_. (b) Mo 3d XPS spectrum after PE, displaying the dominant Mo^4+^ doublet along with higher binding energy components (Mo^6+^) attributed to oxidized Mo species.

To examine excitation power–dependent phonon behavior in the PE ML, Figure 7a shows Raman spectra illustrating the evolution of the E_2g_^1^ and A_1g_ modes with increasing laser power, with the measurement location indicated in Figure 7b. As the excitation power increases from 0.5 μW to 12.5 mW, both modes exhibit a gradual red shift accompanied by enhanced Raman intensity (Figure 7d), consistent with laser-induced local heating, phonon softening, and reduced heat dissipation in oxide-protrusion-covered regions.^58^ Beyond ∼12.5 mW (up to 50 mW), the trend reverses, with both modes showing a progressive blue shift and reduced intensity (Figure 7d), indicating laser-induced modification of the exposed region and relaxation of tensile strain associated with oxide protrusions. Optical images before and after high-power exposure (Figure 7b,c) reveal local brightening, confirming this transition. Despite these changes, the persistence of strong E_2g_^1^ and A_1g_ modes indicates that the underlying MoS_2_ lattice remains structurally intact. The extracted peak positions and intensities (Figure 7e,f) demonstrate a threshold-dependent response, where low-power excitation induces thermal effects, while higher power leads to strain relaxation and reduced Raman scattering.

**Figure 7:**
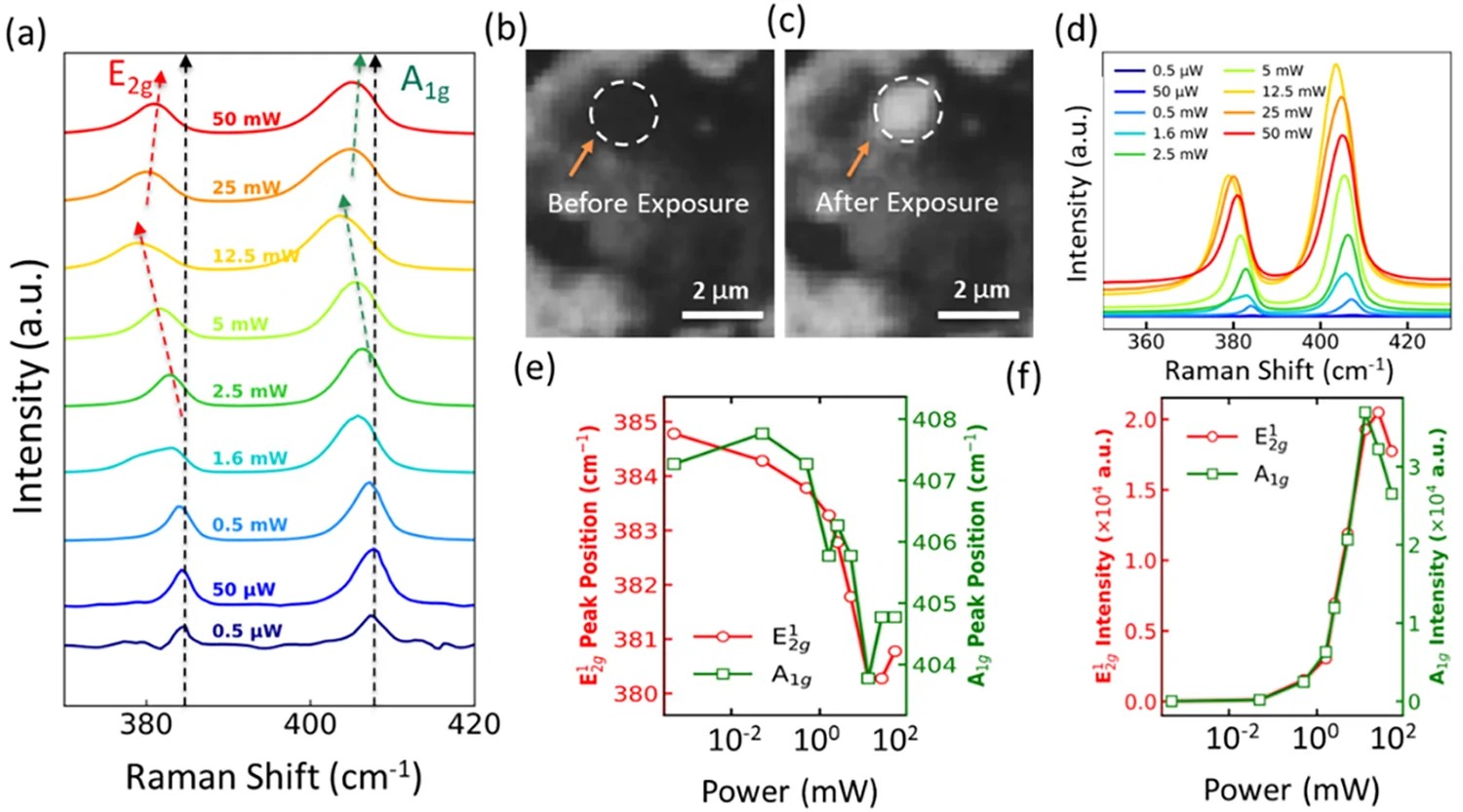
(a) Power-dependent Raman spectra acquired from the PE ML containing oxide protrusions, corresponding to the area encircled in (b), recorded before and (c) after laser exposure. (d) Power-dependent Raman intensities, extracted from the same spectra shown in (a) correlating with the phonon shifts observed in (a). (e) Extracted peak positions of the E_2g_^1^ and A_1g_ modes from (a) as a function of excitation power. (f) Extracted peak intensities of the E_2g_^1^ and A_1g_ modes from (d) as a function of excitation power.

To further probe nanoneedle–induced strain effects on vibrational modes, Raman spectra were extended to 2000 cm^–1^, revealing an additional mode at ∼1439 cm^–1^. Figure 8a compares spectra acquired at 5 mW from the PE ML (red), ME ML (green), and a bulk region (orange). The exclusive appearance of the 1439 cm^–1^ mode in the PE ML, and its absence in both ME and bulk samples, indicates its origin from a surface-modified phase formed during the PE process. Power-dependent Raman measurements (Figure 8b) show that this feature is absent at low excitation powers (0.5 μW–0.5 mW), emerges at ∼1.6 mW, strengthens up to 5 mW, and gradually weakens beyond 12.5 mW before diminishing at higher powers. The persistent E_2*g*_^1^ and A_1g_ phonons throughout this range confirm preservation of the underlying ML lattice. The finite excitation window indicates that the ∼1439 cm^–1^ mode is strain-activated, becoming detectable only under moderate excitation and suppressed at higher powers due to laser-induced modification of the locally strained region. Its attenuation above ∼12.5 mW coincides with optical brightening, blue shifts and intensity reduction of E_2g_^1^ and A_1g_ phonons, evidencing strain relaxation and oxide-protrusion modification. Together, these results establish the ∼1439 cm^–1^ mode as a sensitive spectral marker of the oxide-protrusion–modified ML, stable under moderate excitation while diminishing upon strain relaxation without structural degradation of the ML.

**Figure 8:**
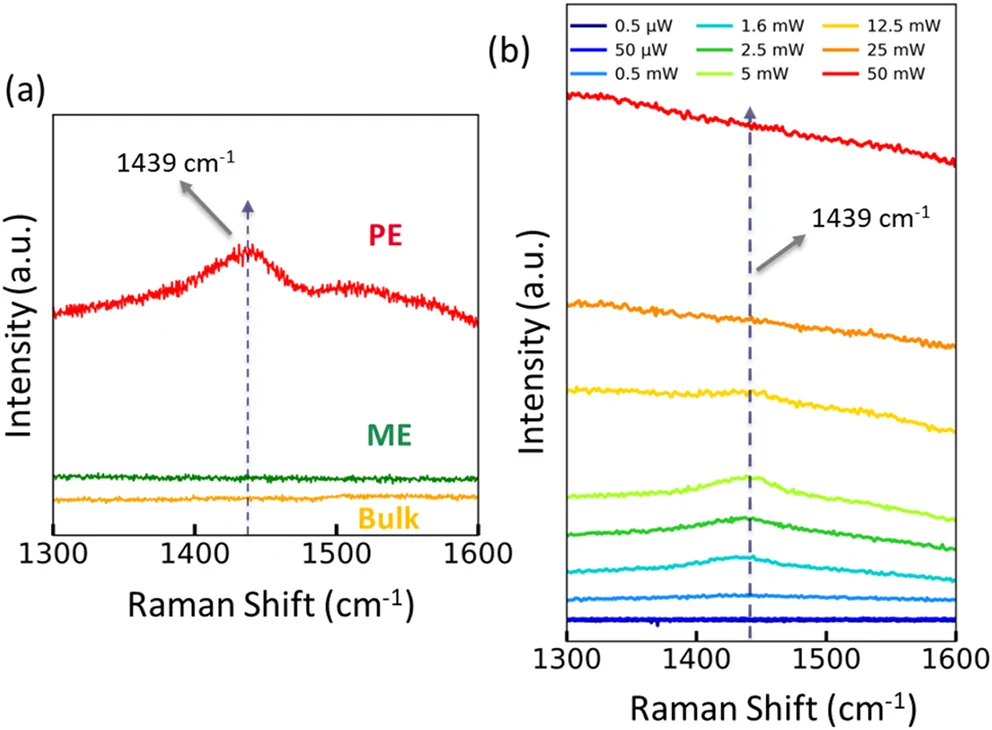
(a) Comparative Raman spectra acquired at a laser power of 5 mW, extended over the 1300–1600 cm^–1^ range, from the PE ML (red), ME ML (green), and a bulk region (orange). (b) Power-dependent Raman spectra acquired from the PE ML, extended over the 1300–1600 cm^–1^ range.

## Conclusion

In conclusion, the photo-oxidation-assisted exfoliation process offers an effective route for strain engineering in monolayer MoS_2_, enabling modulation of excitonic emission. The formation of nanoneedle-like oxide protrusions during exfoliation process locally deforms the lattice, generating spatially varying strain fields that play a dominant role in governing the excitonic response. Correlated PL and Raman measurements establish a clear relationship between strained regions, exciton peak shifts, and enhanced emission, with the PL intensity increasing by approximately 3-fold on a conductive substrate relative to SiO_2_, accompanied by a red shift of the in-plane vibrational mode consistent with strain. Additionally, emergent novel near-infrared PL emission features observed in selected monolayers and unexfoliated regions indicate strain-modified local electronic environments. Overall, the intrinsically formed oxide nanoneedles establish a self-generated strain-engineering platform that, unlike conventional approaches requiring external deformation, specialized substrate prepatterning, or complex heterostructures, enables tunable and enhanced excitonic emission while extending the optical response of monolayer TMDCs into the near-infrared region.

## Supplementary Material



## Notes

AFM characterization of nanoneedle structures in Samples 2 and 3; representative 2D AFM topographic images; P1 and P2 line profiles; nanoneedle height and lateral-dimension analysis (Figure S1); AFM surface-coverage analysis of nanoneedle structures in Sample 1; height-thresholded AFM topography; projected surface-coverage analysis (Figure S2) (PDF)
