# Supplementary material for: Self-Assembled Oxide Nanoneedle-Induced Strain for Tunable Excitonic Emission in Monolayer MoS2
Source: ACS Omega. 2026 Oct 2;11(41):62866–76. doi: 10.1021/acsomega.6c04805 (PMC13688502; doi:10.1021/acsomega.6c04805)
Supplement: Supplementary file 1 [file ao6c04805_si_001.pdf]

SUPPORTING INFORMATION

Self-assembled oxide nano-needle-induced strain for tunable excitonic emission in monolayer MoS_2

*Rashed H. Lone [1,2] * Shreya Gaonkar[2], Deepak Sharma[1], Shruti Nirantar[1] and E. S. Kannan[2,3] **

[1]School of Engineering, STEM College, RMIT University, Melbourne, VIC 3001, Australia
[2]Department of Physics, BITS-Pilani K. K. Birla Goa Campus, Zuarinagar, Goa-403726, India.
[3]Department of Electrical and Electronic Engineering, BITS-Pilani K. K. Birla Goa Campus, Zuarinagar, Goa 403726, India
*email: rashedlone55@gmail.com, Phone: 61 0451975785

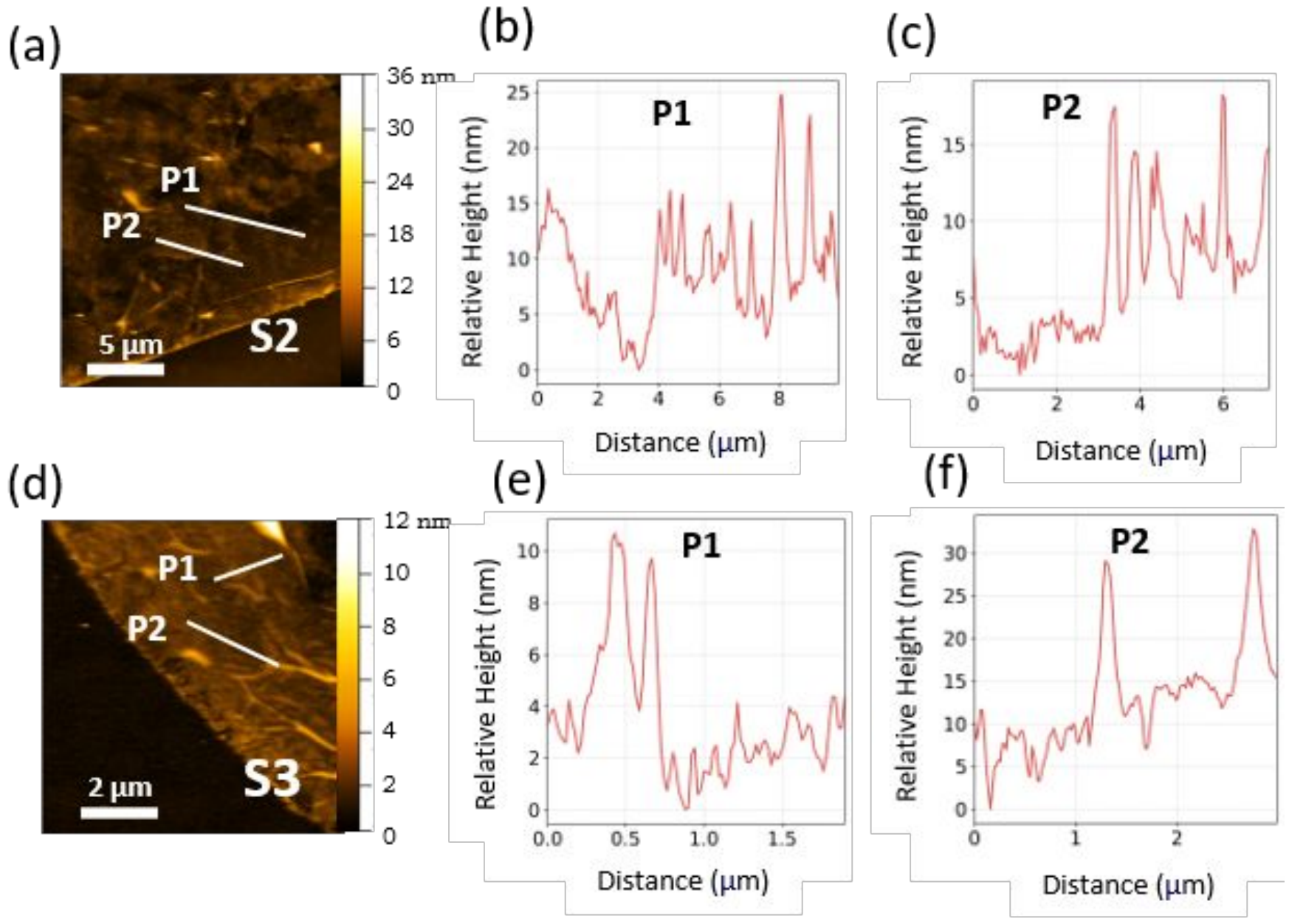

Figure S1. *AFM characterization of nano-needle structures in Samples 2 and 3. (a) Sample 2 AFM topography; (b,c) corresponding P1 and P2 line profiles; (d) Sample 3 AFM topography; (e,f) corresponding P1 and P2 line profiles. The profiles provide nano-needle height and lateral-dimension measurements.*

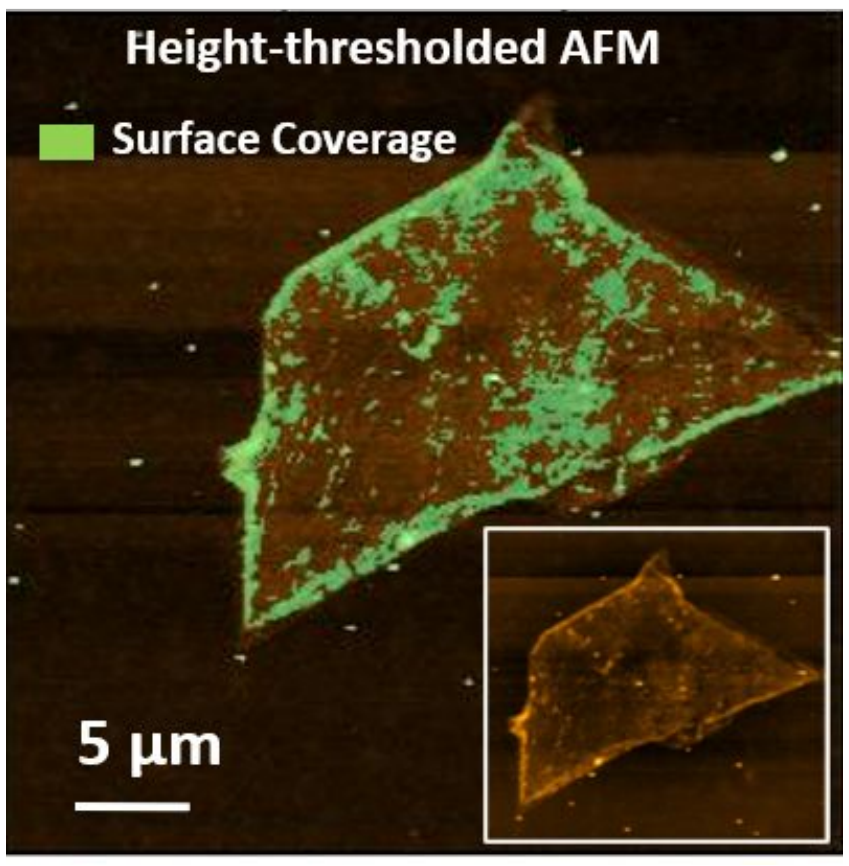

Figure S2. *Surface-coverage analysis of nano-needle structures. Height-thresholded AFM topography of Sample 1, with the highlighted regions (green) representing areas exceeding a height threshold of approximately 2–5 nm, attributed to the nano-needle structures. The original AFM topography is shown in the inset. The corresponding projected surface coverage is 27.23%.*
